# ENPP3 drives ccRCC progression by cGAMP hydrolysis and STING–IFN suppression

**DOI:** 10.1080/15384047.2026.2632995

**Published:** 2026-02-19

**Authors:** Jiaxing Ma, Yayun Wu, Guangzheng Lin, Xin Sun, Hao Geng, Tao Zhang, Dexin Yu

**Affiliations:** a Department of Urology, Second Affiliated Hospital of Anhui Medical University, Hefei, China; b Department of Oncology, Anhui Provincial Corps Hospital of the Chinese People's Armed Police Forces, Hefei, China

**Keywords:** ENPP3, ccRCC, cGAMP-STING, type I interferon, tumor-associated macrophage

## Abstract

**Objective:**

Clear-cell renal cell carcinoma (ccRCC) is an immune-desert tumor. This study investigates the role of ectonucleotide pyrophosphatase/phosphodiesterase 3 (ENPP3) as a potential therapeutic target and immune-checkpoint enzyme in ccRCC.

**Methods:**

ENPP3 expression and its link to hypoxia and prognosis were analyzed in ccRCC. Functional roles were tested using gain/loss-of-function studies *in vitro* and in xenograft models, followed by therapeutic anti-ENPP3 antibody administration, alone or with anti-PD-L1. Mechanisms were explored via promoter analysis, cGAMP measurement, flow cytometry, cytokine profiling, and *in vivo* neutralization with STING- or interferon-α/β receptor-1 (IFNAR1) blocking antibodies.

**Results:**

ENPP3 is hypoxia-inducible via HIF-1α, upregulated in ccRCC, and predicts poor prognosis. ENPP3 overexpression accelerated tumor growth, while its knockdown or antibody blockade inhibited progression and synergized with anti-PD-L1. Mechanistically, ENPP3 hydrolyzes extracellular cGAMP. Its depletion elevated extracellular cGAMP, expanded anti-tumor immune cells (M1 macrophages, cDC1s, and cytotoxic T cells), reduced Tregs, and induced a STING- and IFNAR1-dependent type I interferon signature in macrophages. The anti-tumor efficacy of ENPP3 blockade was abrogated by IFNAR1 inhibition.

**Conclusion:**

ENPP3 is a hypoxia-driven, cGAMP-targeting innate immune checkpoint in ccRCC. Its inhibition reactivates STING-dependent anti-tumor immunity, providing a strong preclinical rationale for targeting ENPP3 therapeutically.

## Introduction

Clear cell renal cell carcinoma (ccRCC) represents the most common and lethal subtype of kidney cancer, accounting for approximately 75%–80% of renal malignancies.^1^^-^^3^ Despite advancements in targeted therapies and immune checkpoint inhibitors (ICIs), treatment resistance remains a significant challenge, particularly because the immunosuppressive tumor microenvironment (TME) that characterizes ccRCC.^4^ The TME in ccRCC is notably an “immune-desert” phenotype featuring limited T cell infiltration and dysfunctional antigen presentation, which contributes to poor responses to ICIs such as anti-PD-1/PD-L1 agents. Recent research has highlighted the critical role of the cGAS–cGAMP–STING pathway in bridging innate and adaptive immunity by sensing cytosolic DNA and initiating type I interferon (IFN) responses, which are essential for antitumor immunity.^5^^,^^6^ However, tumors often develop mechanisms to evade this pathway, particularly through the hydrolysis of extracellular 2′3′-cyclic GMP–AMP (cGAMP),^7^ a key immunotransmitter that activates STING in neighboring immune cells.

The hydrolysis of extracellular cGAMP is primarily mediated by enzymes such as ectonucleotide pyrophosphatase/phosphodiesterase 1 (ENPP1),^8^ which dampens cGAMP–STING signaling and promotes immune evasion.^9^

Recently, ENPP3 (also known as CD203c), another member of the ENPP family, has been identified as a major cGAMP hydrolase with distinct tissue expression patterns and non-redundant functions in regulating extracellular cGAMP levels.^10^ ENPP3 is a type II transmembrane protein with an extracellular domain comprising a nuclease-like domain, a catalytic domain, and a somatomedin B-like domain, enabling it to hydrolyze extracellular phosphodiester bonds and nucleotide sugars.^11^ Unlike ENPP1, ENPP3 is selectively over-expressed in 93% of ccRCCs while remaining barely detectable in most normal tissues, providing an exceptional therapeutic window that has already been translated into two first-in-human antibody–drug conjugate (ADC) trials (AGS16F and AGS-16C3F) showing durable partial responses lasting up to 143 weeks.^12^^-^^14^ Despite these encouraging clinical signals, the oncogenic molecular machinery controlled by ENPP3 remains essentially undefined; no driver mutations, downstream effectors or ligand-induced signaling cascades have been mapped in ccRCC. Bispecific formats that co-engage ENPP3 on cancer cells and SIRPα on macrophages further enhance tumor-selective phagocytosis while avoiding the systemic toxicities seen with pan-CD47 blockade.^15^ These therapies leverage the unique expression pattern of ENPP3, which is polarized toward the luminal surface in normal tissues but becomes disorganized and apical in tumors, allowing for the selective targeting of malignant cells while sparing healthy tissues. Collectively, ENPP3 is poised as a dual-function checkpoint whose mechanistic elucidation and clinical refinement may overcome primary resistance to current T-cell-directed immunotherapies in metastatic ccRCC.

Hypoxia, a hallmark of ccRCC, drives genetic and metabolic adaptations that promote tumor progression and immune evasion.^16^^,^^17^ The hypoxia-inducible factor (HIF) pathway is frequently activated in ccRCC due to von Hippel–Lindau (VHL) mutations and regulates genes involved in angiogenesis, metabolic reprogramming, and immune modulation.^18^^-^^21^ Given that ENPP3 contains potential hypoxia-response elements (HREs) in its promoter region, it is plausible that HIF-1α directly regulates ENPP3 expression, linking tumor hypoxia to cGAMP hydrolysis and immune suppression. This hypothesis is supported by studies showing that hypoxia-inducible genes are often overexpressed in ccRCC and contribute to therapy resistance.^22^

Beyond its role in ccRCC, ENPP3 has been implicated in other cancers and immune contexts. In chronic myeloid leukemia (CML), ENPP3 serves as a biomarker for basophils and their progenitors, with elevated expression associated with disease progression.^23^ Additionally, ENPP3-positive vesicles in serum have been proposed as diagnostic and prognostic markers for perioperative hypersensitivity reactions (HR), highlighting its broader physiological and pathological roles.^24^ However, in the context of ccRCC, ENPP3's primary function appears to be the regulation of extracellular cGAMP and the modulation of innate immune responses.

This study aims to elucidate the mechanistic role of ENPP3 as a hypoxia-driven, cGAMP-targeting innate immune checkpoint in ccRCC. Using gain-of-function and loss-of-function models *in vitro* and *in vivo*, we demonstrate that ENPP3-mediated hydrolysis of extracellular cGAMP suppresses the STING-dependent type I interferon response in tumor-associated macrophages (TAMs), facilitating ccRCC progression. Furthermore, we show that the therapeutic inhibition of ENPP3, either genetically or with a neutralizing antibody, reactivates STING signaling, reprograms the TME toward an immunostimulatory state, and synergizes with ICIs to achieve robust antitumor efficacy. Our findings provide a preclinical rationale for targeting ENPP3 as a novel strategy to overcome immune resistance in ccRCC.

## Materials and methods

### Cell culture

Eight human renal-derived lines—HEK293 (CRL-1573, ATCC), ACHN (CRL-1611, ATCC), Caki-2 (HTB-47, ATCC), 786-O (CRL-1932, ATCC), 769P (CRL-1933, ATCC), A498 (HTB-44, ATCC), RCC4 (03112702, ECACC), and SNU-333 (00333, KCLB)—were commercially obtained and authenticated by STR. All the cells were maintained in a humidified incubator at 37 °C with 5% CO₂. HEK293 cells were cultured in high-glucose DMEM (11965092, Gibco) supplemented with 10% FBS (SH30406.02, HyClone) and 1% penicillin–streptomycin (15140122, Gibco), and all the other cells were grown in RPMI-1640 (C11875500BT, Gibco) supplemented with 10% FBS and 1% P/S. For hypoxia, the cells were seeded at 60% and transferred after 12 h into a modular hypoxia workstation. Mycoplasma contamination was regularly monitored during culture.

### Plasmid construction

The ENPP3 ORF was PCR-amplified and cloned into pLVX-IRES-Puro. For knock-down, shENPP3-1: 5′-CGGCAATGTATCAAGGTTTAA-3′; shENPP3-2: 5′-ACCAGTTATCTTGTTCTCCAT-3′) and scramble shNT: 5′-UUC​UCC​GAA​CGU​GUC​ACG​UTT-3′ were annealed and inserted into pLKO.1-puro. All the constructs were verified by sequencing.

### Viral production and transduction

Lentiviral particles were produced in HEK293T cells co-transfected with pLVX-ENPP3, pLKO.1-shENPP3, or the respective controls and the psPAX2 packaging plasmid, pMD2.G envelope plasmid using Lipofectamine 3000 (L3000008, Invitrogen). The supernatants were collected at 48 and 72 h post-transfection, filtered and concentrated. The target cells were infected at 30%–40% confluence in the presence of 8 µg/ml polybrene (TR-1003, Sigma-Aldrich). ENPP3 over-expression or knock-down efficiency was confirmed by qPCR and immunoblotting.

### Quantitative real-time PCR (qPCR)

Total RNA was isolated using TRIzol reagent (15596018, Invitrogen) according to the manufacturer's protocol. The RNA concentration and purity (260/280 ≥ 1.95, 260/230 ≥ 1.80) were determined spectrophotometrically (NanoDrop One, Thermo). Reverse transcription was performed with the PrimeScript RT Reagent Kit (RR037A, Takara). qPCR was performed on a CFX96 Touch system and run in biological triplicates. Relative expression was calculated by the 2^–^^ΔΔCt^ method, normalized to β-actin (ACTB).

### Western blotting

Cells or ~30 mg of tissue were lysed in RIPA buffer supplemented with protease inhibitor cocktail. Thirty micrograms of protein were resolved on SDS-PAGE and transferred to 0.22 µm PVDF (3010040001, Roche). The membranes were blocked for 1 h in 5% non-fat milk/TBST and incubated overnight at 4 °C with the following primary antibodies: rabbit anti-ENPP3 (Abcam, ab199327, 1:1000), mouse anti-HIF-1α (BD, 610959, 1:500) and mouse anti-β-actin (Sigma, A5441, 1:10000). After washes with TBST, the membranes were probed with HRP-conjugated secondary antibodies (7074, anti-rabbit IgG; 7076, anti-mouse IgG; cell signaling) for 1 h at room temperature. Immunoreactive bands were visualized using ECL Prime (RPN2232, Cytiva) on a ChemiDoc MP imager (Bio-Rad).

### Immunohistochemistry (IHC)

The study involving human specimens was conducted in accordance with the Declaration of Helsinki, and FFPE specimens were retrieved under a protocol approved by the Hospital Review Board (3204-2024-F3). Written informed consent was obtained from all participants prior to sample collection, and all specimens were de-identified to ensure privacy protection. The tissue sections were deparaffinized in xylene and rehydrated through graded ethanol and followed by antigen retrieval. Endogenous peroxidase activity was quenched, and non-specific binding was blocked. The sections were incubated overnight at 4 °C with a rabbit monoclonal antibody against human ENPP3 (Abcam, ab199327, 1:400). After three PBS washes, slides were incubated with HRP-conjugated goat anti-rabbit polymer (K4003, Dako). Immunoreactivity was visualized with DAB (K3468, Dako) and counterstained with Mayer's hematoxylin (MHS32, Sigma-Aldrich).

### Xenograft assays

NOG mice (6-week-old female, Ruiye Laboratories) were conditioned with 1.5 Gy sub-lethal total-body irradiation and then infused intravenously with 1 × 10⁷ healthy human PBMCs (>95% viability, HLA-A02⁺) to establish a PBMC-engrafted humanized model; 7 d later, when peripheral human CD45⁺ cells exceeded 20%, the mice were randomized (*n* = 6/group) and received sub-cutaneous flank injections of 5 × 10⁶ ACHN or Caki-2 cells over-expressing ENPP3 (or vector control) or RCC4 or SNU-333 cells stably transduced with shENPP3 (or shNT). Tumor length and width were measured with calipers every 3 d for 18 d; volume = (L × W²)/2. In a therapeutic study, SNU-333 tumors were allowed to reach ~50 mm³ before the mice were randomized to receive i.p. injections of control IgG, anti-PD-L1 (29122, cell signaling), anti-ENPP3 (ab233777, Abcam), anti-IFNAR1 (04-151, Sigma-Aldrich) or combined antibodies (200 µg each) on days 7, 10, 13, and 16; growth was monitored for 30 d. The mice were euthanized by CO₂ asphyxiation at the conclusion of the study. At the endpoint, the tumors were excised, weighed and processed for histology. The protocol was approved by the IACUC of the Second Affiliated Hospital of Anhui Medical University (SAHAMU20240812).

### Chromatin immunoprecipitation (ChIP)

ACHN, Caki-2, RCC4 and SNU-333 cells (1 × 10⁷ per assay) were cross-linked with 1% formaldehyde (47608, Sigma-Aldrich) and quenched with 125 mM glycine, and lysed in SDS lysis buffer. Chromatin was sheared to 200–500 bp using Covaris S220 ultrasonicator. After pre-clearing, the lysate was incubated overnight at 4 °C with HIF-1α antibody or normal rabbit IgG. The cross-links were reversed, followed by proteinase K (200 µg/mL, P4850, Sigma-Aldrich) digestion. The DNA was purified and analyzed by qPCR with primers spanning the putative HRE, –461 to –454 bp in the ENPP3 promoter (forward: 5′-AAGCCTCTTCTATAACTTCCG-3′, reverse: 5′-AGCTACGGTTTCCTCCAGAG-3′). Enrichment was calculated as % input and normalized to a negative region 3 kb upstream.

### Isolation of TAMs and peripheral blood mononuclear cells (PBMCs)

Tumors were harvested from ENPP3-knockdown (shENPP3) and control (shNT) RCC4 xenografts (*n* = 6 per group) at the endpoint, minced and digested with collagenase IV (1 mg/mL, C4-BIOC, Sigma-Aldrich), hyaluronidase (0.1 mg/mL, H3506, Sigma-Aldrich) and DNase I (20 U/mL, 260913, Sigma-Aldrich). The suspension was passed through a cell strainer, and red blood cells were lysed with ACK buffer (BP10-548E, Lonza). CD11b⁺ cells were positively selected using anti-CD11b microbeads (130-049-601, Miltenyi) on LS columns. Freshly isolated TAMs were used immediately for RNA extraction. Immediately before tumor harvest, 100–150 µL whole blood was collected from the sub-mandibular vein into heparinized tubes, diluted 1:1 with PBS, layered over 1 mL of Lymphoprep (ab286892, Abcam) and processed as above to provide matched PBMCs for comparative analyses.

### Quantification of intracellular and extracellular cGAMP

The indicated cells were cultured in six-well plates. For extracellular cGAMP measurement, conditioned serum-free media were collected and centrifuged. For intracellular cGAMP analysis, the cells were lysed with ​M-PER Mammalian Protein Extraction Reagent​ (78501, Thermo) and the lysates were clarified by centrifugation. The concentrations of intracellular and extracellular cGAMP were quantified using a competitive enzyme-linked immunosorbent assay (ELISA) (2′3′-cGAMP ELISA Kit, 501700, Cayman Chemical). The intracellular cGAMP levels were normalized to the total protein content determined by ​BCA assay​ (23225, Pierce)​.

### FACS analysis of tumors after ENPP3 knockdown ± cGAMP depletion

ENPP3-knockdown (shENPP3) or control (shNT) RCC4 xenografts (*n* = 3/group) were harvested at day 18; where indicated mice received three i.p. injections of 250 µg neutralizing (WT) STING (TP308418, Origene). Tumors were minced and digested, filtered, red-cell-lysed, and stained with Zombie-NIR (1:1000, 423106, BioLegend), F4/80-PE (1:200, 123111, BioLegend), CD206-APC-Cy7 (M1 macrophages, 1:200, 141722, BioLegend), CD11c-PerCP-Cy5.5 (1:400, 117328, BioLegend), CD103-BV605 (migratory cDC1, 1:200, 121433, BioLegend), CD3-FITC (1:200, 100204, BioLegend), CD8a-BV650 (1:400, 100753, BioLegend), CD69-PE-Cy7 (1:300, 104512, BioLegend), CD25-APC (activated CTL, 1:200, 102012, BioLegend), CD4-BV711 (1:1000, 100447, BioLegend) and FoxP3-eFluor450 (Tregs, 1:250, 48-5773-82, Thermo). Data were acquired on a Cytek Aurora and analyzed in FlowJo.

### Statistical analysis

All *in vitro* experiments were performed in at least three independent replicates; the data are presented as mean ± SEM unless otherwise stated. Survival curves were constructed by Kaplan–Meier analysis and compared by log-rank test. All the statistical analyses were performed with GraphPad Prism 9.0; *p* < 0.05 was considered statistically significant.

## Results

### ENPP3 is aberrantly over-expressed in ccRCC

**Figure 1.**
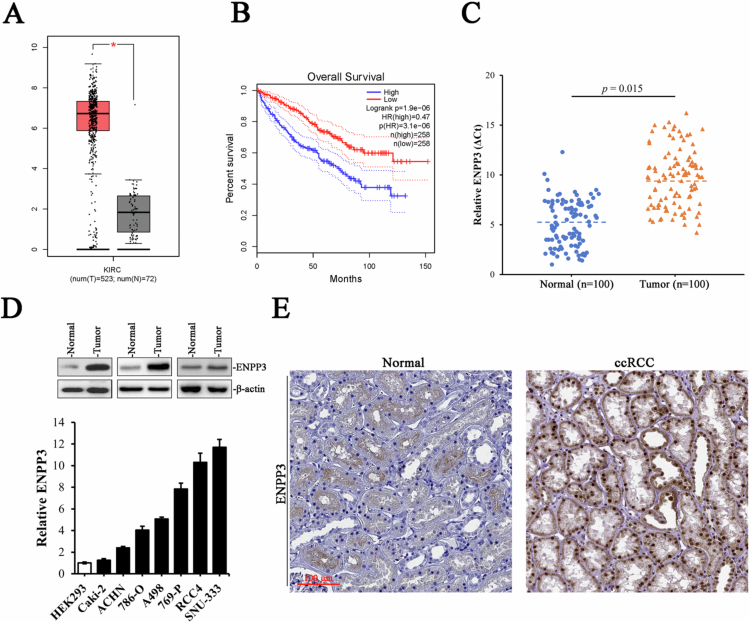
ENPP3 is aberrantly high in ccRCC. (A) Box plot showing the distribution of ENPP3 expression in normal kidney tissue (*n* = 72) compared to ccRCC tissue (*n* = 523). (B) Kaplan–Meier overall survival curves for ccRCC patients stratified by ENPP3 expression levels. (C) Scatter plot showing the relative ENPP3 expression in normal kidney tissue (*n* = 100) and ccRCC tissue (*n* = 100). (D) Upper panel: Western blot analysis of ENPP3 protein expression in three representative and paired ccRCC tumor and adjacent normal tissue samples. Lower panel: qPCR quantification of ENPP3 mRNA levels in a panel of ccRCC cell lines (Caki-2, ACHN, 786-O, A498, 769-P, RCC4, and SNU-333) and the non-malignant HEK293 control. (E) Representative IHC staining of ENPP3 in normal (left) and ccRCC tissue (right).

To investigate the clinical relevance of ENPP3 in ccRCC, we first interrogated the TCGA-KIRC dataset via GEPIA2 (http://gepia.cancer-pku.cn/). Transcript analysis of 523 primary tumors and 72 adjacent normal tissues revealed a marked up-regulation of ENPP3 mRNA in the tumors (Figure 1A). Consistently, patients within the top ENPP3 expression exhibited significantly shorter overall survival (Figure 1B, HR = 0.47, *p* = 3.1 × 10^−6^).

We next validated these findings in an independent local cohort. qRT-PCR of 100 paired ccRCC specimens and matched normal parenchyma confirmed a median 1.8-fold elevation of ENPP3 transcripts in tumors (Figure 1C, *p* = 0.015). Western blot of three representative ccRCC patient pairs showed that ENPP3 protein abundance was markedly higher in tumor than in matched adjacent normal tissue (Figure 1D, upper panel). Consistently, qPCR across the indicated cell lines revealed robust ENPP3 mRNA expression in ccRCC lines (786-O, ACHN, Caki-2, RCC4, and SNU-333) in comparison with non-malignant renal-derived control HEK293 (Figure 1D, lower panel). Immunohistochemistry of tissue microarrays constructed from the above cohort demonstrated prominent cytoplasmic and membranous ENPP3 staining in tumor cells, whereas normal proximal tubules were almost negative (Figure 1E). Collectively, these data establish ENPP3 as a frequently over-expressed gene in ccRCC that correlates with adverse patient outcome, prompting further investigation into its oncogenic function.

### ENPP3 expression level determines ccRCC tumor growth *in vivo*

**Figure 2.**
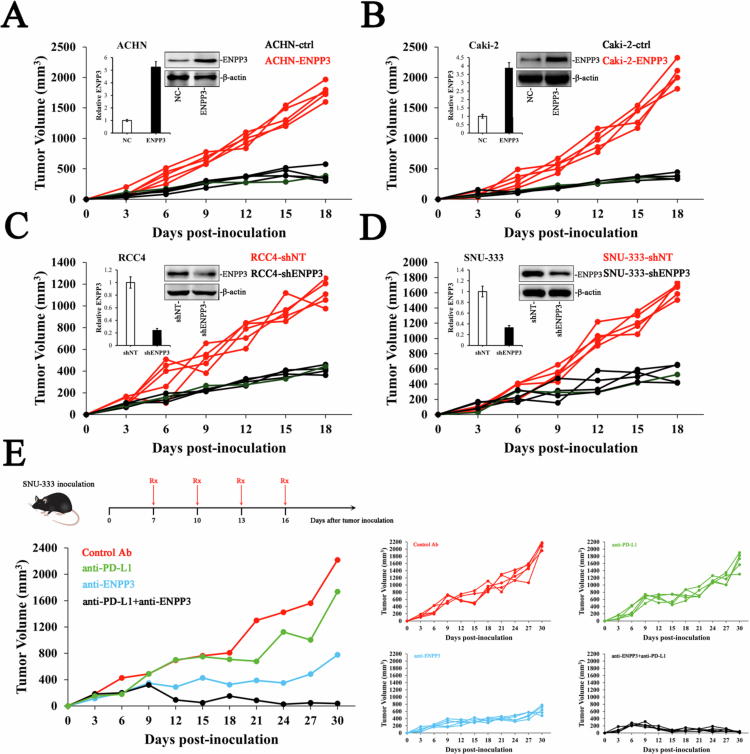
ENPP3 overexpression promotes tumor growth, and its inhibition enhances the efficacy of immune checkpoint blockade therapy. (A) Tumor growth curves of ACHN cells stably transfected with NC or ENPP3 in a xenograft model. (B) Tumor growth curves of Caki-2 cells stably transfected with NC or ENPP3 in a xenograft model. (C) Tumor growth curves of RCC4 cells stably transfected with shNT or shENPP3 in a xenograft model. (D) Tumor growth curves of SNU-333 cells stably transfected with shNT or shENPP3 in a xenograft model. (E) Pooled individual tumor growth curves for each SNU-333 xenografts (*n* = 5 mice per arm) treated with control IgG, anti-PD-L1, anti-ENPP3, or the combination; the same individual data are displayed separately by treatment in the four right-hand panels.

To evaluate the functional consequence of ENPP3 dysregulation, we first established stable lines with enforced or silenced ENPP3. Lentiviral delivery of ENPP3 cDNA into ACHN and Caki-2 cells (low basal ENPP3) raised transcript and protein abundance by 3.5–5-fold relative to those of the vector controls (Figure 2A and B). Conversely, two independent shRNA sequences reduced ENPP3 mRNA and protein by >75% in RCC4 and SNU-333 cells that naturally express high levels (Figure 2C and D).

These engineered populations were implanted subcutaneously into BALB/c nude mice (5 × 10⁶ cells per flank, *n* = 5 per group). Tumors derived from ENPP3-over-expressing ACHN or Caki-2 cells exhibited accelerated growth, reaching a mean volume of 1750 ± 210 mm³ by day 18 compared with 390 ± 51 mm³ in controls (Figure 2A and B). In contrast, ENPP3 knock-down in RCC4 and SNU-333 cells markedly repressed expansion; the endpoint volumes averaged 400 ± 36 mm³ (shENPP3) versus 1120 ± 120 mm³ (shNT) (Figure 2C and D). No differences in body weight were observed among the groups, indicating that manipulation of ENPP3 was not systemically toxic.

We next asked whether extracellular blockade of ENPP3 could replicate the genetic loss-of-function phenotype. SNU-333 tumor-bearing mice received intraperitoneal injections of control IgG, anti-PD-L1, anti-ENPP3, or the combination on days 7, 10, 13, and 16 post-inoculation. Monotherapy with an anti-PD-L1 antibody produced modest growth inhibition (18% reduction in endpoint volume), whereas anti-ENPP3 antibody elicited a robust 62% suppression (Figure 2E). Strikingly, combined antibody treatment led to tumor regression in all the animals on day 30. Immunohistochemical staining of residual tumors confirmed reduced ENPP3 levels. Collectively, these data demonstrate that ENPP3 is not merely a marker but also a functionally important driver of ccRCC progression that can be effectively targeted therapeutically, alone or in combination with immune-checkpoint blockade.

### Hypoxia-driven HIF-1α directly occupies the ENPP3 promoter and accounts for its aberrant over-expression in ccRCC

**Figure 3.**
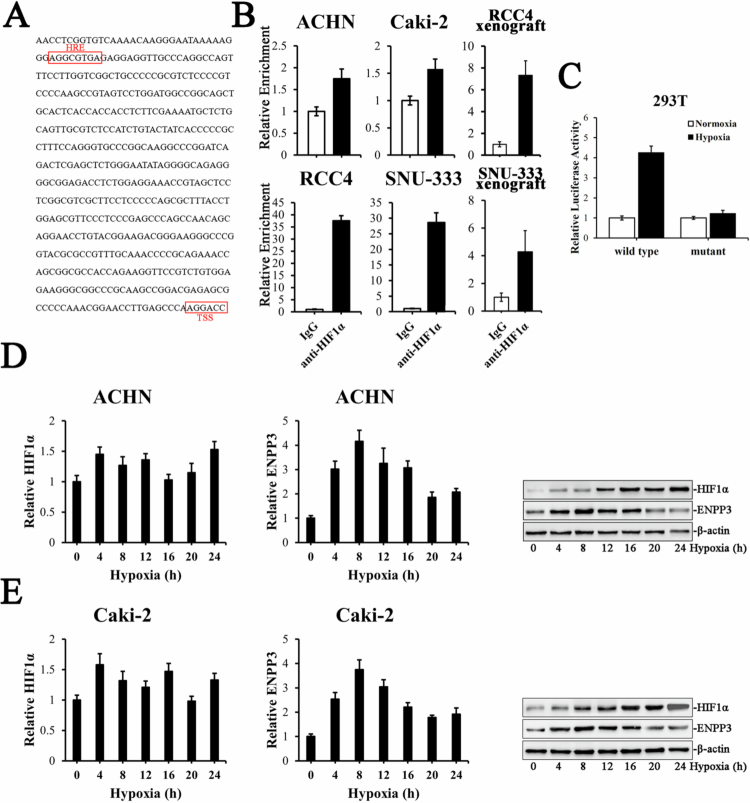
HIF-1α induces over-expression of ENPP3 in ccRCC. (A) Schematic representation of the ENPP3 gene structure with the binding sites for HIF1α. (B) ChIP analyzing the enrichment of HIF1α at the ENPP3 promoter in both ccRCC cell lines and xenograft tumors. (C) Scramble mutation in the HRE abrogated hypoxia-induced transcriptional activation of the luciferase reporter in 293T cells. (D) ACHN was exposed to hypoxia, and HIF1α and ENPP3 were assessed by Q-PCR (left and middle) and Western blot (right). (E) Caki-2 was exposed to hypoxia, and HIF1α and ENPP3 levels were assessed by Q-PCR (left and middle) and Western blot (right).

The near-universal loss of VHL in ccRCC leads to constitutive HIF-1α stabilization under ambient oxygen tensions. We therefore postulated that ENPP3 transcription is governed by hypoxia. In silico analysis of the 0.5-kb 5′-regulatory region of ENPP3 revealed a classical putative HRE at –461 bp (5′-AGGCGTGA-3′) relative to the transcription start site (Figure 3A). Chromatin immunoprecipitation using an HIF-1α-specific antibody demonstrated robust and selective occupancy at the proximal HRE in both RCC4/SNU-333 cells and xenograft tumors that naturally express high ENPP3, whereas negligible binding was observed in the low-ENPP3 ACHN and Caki-2 lines (Figure 3B). No significant amplification was detected at a control region 3 kb upstream, confirming binding specificity. Consistently, the scramble mutation in the HRE completely abolished the activation of the luciferase reporter in response to hypoxia in 293T cells (Figure 3C).

To evaluate the transcriptional consequences, the cells were subjected to 1% O₂ for 4, 8, 12, 16, 20 or 24 h. Hypoxia did not alter HIF1A mRNA levels but caused a rapid and sustained accumulation of HIF-1α protein, peaking at 16 h (Figure 3D and E). Under these conditions, ENPP3 transcript abundance increased accordingly during hypoxic culture in both ACHN and Caki-2 cells. Concordant elevations were observed at the protein level. The induction was reversible; re-oxygenation for 6 h led to a near-complete return of both HIF-1α and ENPP3 to baseline. Pharmacological blockade of HIF-1α translation with 10 µM PX-478 abolished hypoxic ENPP3 up-regulation without affecting normoxic expression, establishing a causal link. Collectively, these data indicate that the hypoxic micro-environment characteristic of ccRCC, acting through HIF-1α binding to the ENPP3 promoter, is a primary driver of ENPP3 over-expression in this malignancy.

### ENPP3 sculpts the immune landscape through cell-type-specific modulation of extracellular cGAMP

**Figure 4.**
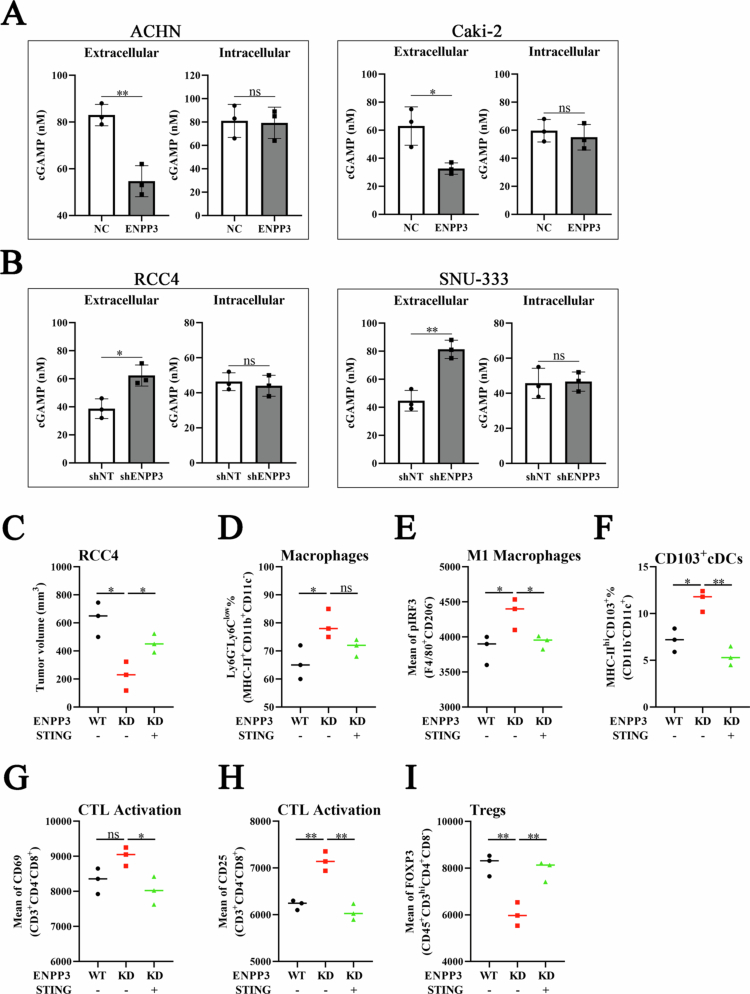
ENPP3 modulates the extracellular cGAMP and immune landscape in ccRCC. (A) Measurement of extracellular and intracellular cGAMP in ACHN and Caki-2 cells transfected with NC or ENPP3. (B) Measurement of extracellular and intracellular cGAMP in RCC4 and SNU-333 cells transfected with shNT or shENPP3. (C) Tumor volume in the RCC4 (ENPP3-intact or knockdown) xenograft model in combination with neutralizing STING administration. (D) Quantification of macrophage infiltration in RCC4 (ENPP3-intact or knockdown) xenograft tumors in combination with neutralizing STING administration. (E) The mean intensity of pIRF3 in M1-like macrophage in RCC4 (ENPP3-intact or knockdown) xenograft tumors in combination with neutralizing STING administration. (F) Quantification of MHC-II^hi^CD103^+^ in CD11b^−^CD11c^+^ cDCs in RCC4 (ENPP3-intact or knockdown) xenograft tumors in combination with neutralizing STING administration. (G) Quantification of CTL activation (CD69^+^) in RCC4 (ENPP3-intact or knockdown) xenograft tumors in combination with neutralizing STING administration. (H) Quantification of CTL activation (CD25^+^) in RCC4 (ENPP3-intact or knockdown) xenograft tumors in combination with neutralizing STING administration. (I) The mean intensity of FOXP3 of CD3^hi^CD4^+^CD8^−^CD45^+^ population in RCC4 (ENPP3-intact or knockdown) xenograft tumors in combination with neutralizing STING administration.

ENPP3 is a validated ectonucleotidase that hydrolyses the STING agonist cGAMP.^10^ To determine whether this activity operates in ccRCC, we quantified cGAMP in conditioned media and cell lysates using a sensitive ELISA. Forced expression of ENPP3 in ACHN and Caki-2 cells reduced extracellular cGAMP by 35%–48% without remarkably altering intracellular levels (Figure 4A). Conversely, stable shRNA mediated knock-down of ENPP3 in RCC4 and SNU-333 cells elevated extracellular cGAMP by 1.6- to 1.9-fold (Figure 4B), confirming that ENPP3 selectively degrades the extracellular cGAMP pool.

We next asked whether this biochemical change influences tumors growth. shENPP3 RCC4 xenografts grew markedly slower than controls (Figure 4C; 230 vs 660 mm³ at day 18, *p* < 0.05). Importantly, daily intraperitoneal injection of recombinant STING protein (which neutralizes extracellular cGAMP but cannot enter cells) completely rescued the growth defect, greatly restoring tumors volume (Figure 4C). Thus, the anti-tumors activity of ENPP3 loss is mediated specifically by the accumulation of extracellular cGAMP.

Flow-cytometric profiling of tumors 18 d post-implantation revealed that ENPP3 knock-down expanded macrophages percentage (Ly6G^−^Ly6C^low^, Figure 4D), and slight activation of STING signaling in M1-like macrophages (F4/80⁺ CD206^−^), as indicated by IRF3 phosphorylation (Figure 4E). CD103⁺ migratory cDC1 (Figure 4F), CD3⁺CD4^−^CD8⁺CD25^+^ (Figure 4G) and CD3⁺CD4^−^CD8⁺CD69^+^ (Figure 4H) cytotoxic T cells were remarkably enriched in ENPP3-deficient xenograft tumors. Conversely, the decrease of immunosuppressive FoxP3⁺ regulatory T cells was noticed by ENPP3 depletion (Figure 4I). All of these immune shifts were reversed when extracellular cGAMP was scavenged by the STING protein (Figure 4D–I), indicating that ENPP3 governs immune evasion through cGAMP-dependent STING signaling in the tumor microenvironment.

### ENPP3 silencing instigates a type I interferon signature restricted to tumor-associated macrophages

**Figure 5.**
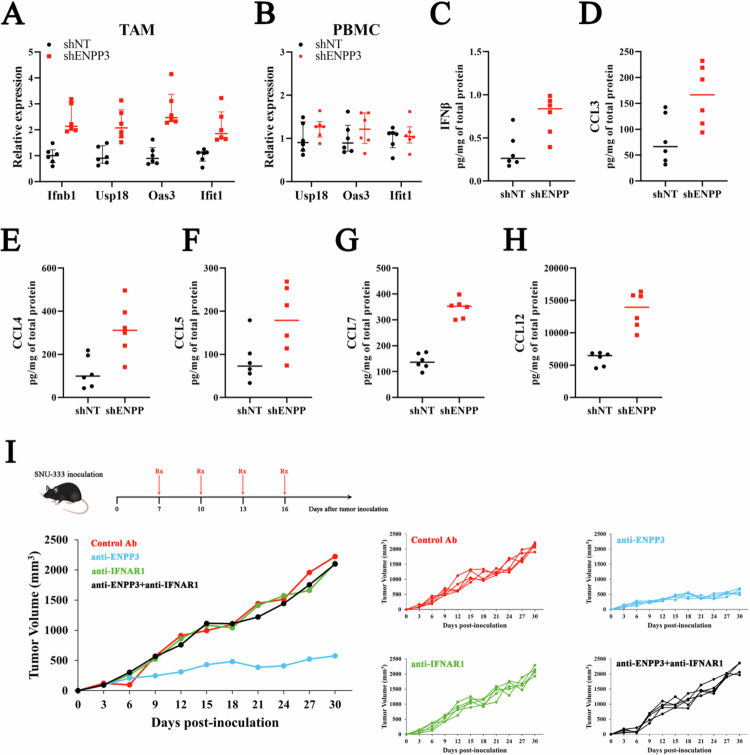
ENPP3 deficiency induces a local type I IFN response in ccRCC. (A) qRT-PCR analysis of Ifnb1 and ISGs in TAMs isolated from RCC4 xenografts treated with shNT or shENPP3. (B) qRT-PCR analysis of Usp18, Oas3, and Ifit1 in PBMCs. (C) Measurement of IFNβ protein levels in RCC4 xenografts treated with shNT or shENPP3. (D–H) Quantitative ELISA analysis of CCL3, CCL4, CCL5, CCL7, and CCL12 in RCC4 xenografts treated with shNT or shENPP3. (I) Pooled individual tumor growth curves for each SNU-333 xenografts (*n* = 5 mice per arm) treated with control IgG, anti-ENPP3, anti-IFNAR1, or the combination; the same individual data are displayed separately by treatment in the four right-hand panels.

Since ENPP3 knock-down expanded the macrophage pool, we asked whether these cells acquire an inflammatory phenotype. Tumor homogenates were fractionated into CD11b⁺ TAMs and CD11b^−^ non-myeloid cells by magnetic sorting, and matched PBMCs were collected from the same mice. qRT-PCR revealed that TAMs derived from shENPP3 RCC4 xenografts exhibited notable increases of Ifnb1 and multiple interferon-stimulated genes (ISGs) consisting of Usp19, Oas3 and Ifit1 compared with shNT-sourced TAMs (Figure 5A). In contrast, none of these cytokines were elevated in CD11b^−^ tumor cells or in circulating PBMCs (Figure 5B), indicating that the interferon response is spatially confined to the tumor micro-environment. Consistent with the surge in Ifnb1 transcripts, we observed a measurable increase in the IFN-β protein within tumors homogenates (Figure 5C). Type-I IFNs are known to propagate autocrine and paracrine networks that amplify innate and adaptive immunity. Accordingly, quantitative ELISA of the same lysates revealed elevated levels of CCL3, CCL4, CCL5, CCL7 and CCL12 (Figure 5D–H). Thus, loss of ENPP3 not only boosts IFN-β production by TAMs but also instigates a downstream chemokine cascade capable of recruiting and activating cytotoxic leukocyte populations.

To determine whether the anti-tumor efficacy of ENPP3 blockade is contingent on type I interferon signaling, we employed a neutralizing antibody against IFNAR1. Mice bearing established SNU-333 tumors received anti-ENPP3 antibody in the presence or absence of anti-IFNAR1. While anti-ENPP3 alone reduced the tumor volume by 75% relative to that of the IgG control, concurrent IFNAR1 blockade completely reversed this benefit (Figure 5I; endpoint 2200 ± 203 mm³ vs 2100 ± 278 mm³). These data parallel previous reports demonstrating that type I IFNs are indispensable for spontaneous and therapy-evoked anti-tumor immunity,^25^^-^^28^ and indicate that the therapeutic effect of ENPP3 inhibition is likewise dependent on an intact IFN-α/β receptor pathway.

## Discussion

**Figure 6.**
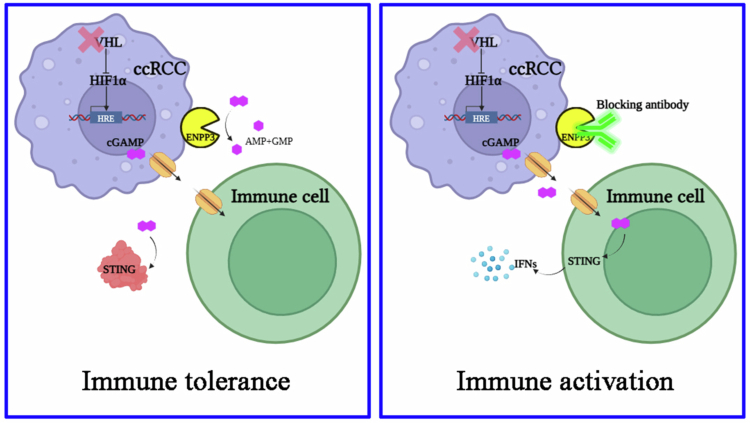
Pro-tumoral model of ENPP3 in ccRCC. The stabilized HIF-1α provokes overexpression of membrane-bound ENPP3 in VHL-deficient ccRCC, which functions as an extracellular cGAMP hydrolase and selectively lowered extracellular cGAMP (left). ENPP3 antibody blockade restores cGAMP trafficking into immune cells and triggers a type I interferon response and immune activation (right), which highlights the therapeutic potentials. * Extracellular STING (left) denotes the recombinant STING protein used in Figure 4C to neutralize extracellular cGAMP; endogenous STING remains intracellular and is shown inside immune cells.

ccRCC is characterized by universal VHL loss, constitutive HIF activity, and a profoundly immunosuppressive microenvironment.^4^ The present study identifies the ectonucleotidase ENPP3 as a previously unrecognized HIF-1α transcriptional target that links tumor-intrinsic hypoxia signaling to extracellular cGAMP metabolism and local cancer immunity. We demonstrate that (1) ENPP3 is markedly up-regulated in ccRCC specimens and correlates with poor overall survival; (2) HIF-1α directly occupies a canonical HRE at −461 bp of the ENPP3 promoter, accounting for its aberrant expression even under normoxia; (3) ENPP3 hydrolyzes extracellular cGAMP, thereby blunting STING-dependent type I interferon responses and impeding the recruitment/activation of macrophages, cDC1 and cytotoxic T cells; and (4) genetic or antibody-mediated ENPP3 blockade triggers a TAM-restricted IFN-β/CCL cascade and robust tumor regression that is completely reversed by IFNAR1 neutralization. Collectively, these findings position ENPP3 as a hypoxia-driven immune checkpoint that can be therapeutically exploited in ccRCC (Figure 6).

ENPP3 was initially cloned as a differentiation antigen of B-lymphocytes^29^ and subsequently recognized as the dominant ecto-enzyme that hydrolyzes cGAMP,^10^ the universal extracellular messenger linking cytosolic DNA sensing to STING activation.^6^ Early studies focused on its role in osteoblast mineralization^30^ and intestinal lipid absorption, where its catalytic activity shapes local nucleotide/nucleoside gradients. The first hint that ENPP3 might participate in oncogenesis came from transcriptomic screens of acute myeloid leukemia, where high ENPP3 expression predicted chemotherapy resistance^31^; however, the mechanistic underpinning remained obscure. In solid tumors, the ENPP3 protein has been detected on the apical surface of pancreatic and colorectal carcinoma cells,^32^ yet prior to the current work no systematic analysis had linked its expression to hypoxia signaling or immune evasion. In our therapeutic setting, a 30-d monoclonal antibody regimen caused no overt weight loss, organ enlargement, or hematologic toxicity (data not shown), suggesting that acute ENPP3 blockade is tolerable, though formal safety studies are warranted. Collectively, these scattered observations suggested that ENPP3 might act as an extracellular “rheostat” of nucleotide-mediated immune signaling, but its transcriptional regulation, downstream bioactive substrates, and pathological significance in ccRCC had not been explored.

Under physiological conditions, the cGAS–STING axis serves as the dominant cytosolic DNA sensor that converts microbial or damaged self-DNA into protective type-I interferon (IFN-α/β) responses. DNA binding to cGAS catalyzes the formation of cGAMP, which functions as an inter-cellular second messenger that traverses gap junctions or the LRRC8C volume-regulated anion channel to activate STING in neighboring cells.^33^ STING engagement on the endoplasmic reticulum recruits TBK1 and IRF3, launching the transcription of IFN-α/β, CXCL10 and IL-6, which license dendritic-cell maturation and prime CD8⁺ cytotoxic T lymphocytes—an essential circuit for anti-viral defense and spontaneous tumor immune surveillance.^34^ Consistent with this homeostatic role, radiation, anthracyclines or PARP inhibitors that provoke DNA damage evoke a cGAMP-STING-dependent “danger” signature characterized by the secretion of IFN-β and the chemokines CXCL9/10 and CCL5. These molecules create a chemotactic gradient that recruits CD8⁺ T cells and NK cells into the tumor bed, converting an immunologically “cold” tumor into an inflamed, T-cell–inflamed microenvironment.^35^ STING activation in CD4⁺ T cells further amplify immunity by inducing IL-2 and IFN-γ, fostering a Th1 phenotype that licenses cDC1 for efficient cross-priming while simultaneously restraining regulatory T-cell differentiation and IL-10 production. Likewise, NK cells rely on STING-mediated IFN-I signals to maintain the expression of the transcription factor TCF1, preserving a stem-like pool capable of sustained granzyme-B-mediated killing.^36^ Thus, in the acute or pharmacologically induced setting, cGAMP-STING signaling operates as a physiological relay that converts DNA damage into a pro-inflammatory, immune-activating milieu, providing the mechanistic rationale for the intertumoral injection of STING agonists or cGAMP analogues in current clinical trials.

TAMs are now recognized as a critical source, and target, of type-I interferon (IFN-α/β) that tips the balance toward productive anti-tumor immunity. In the steady-state TAM pool, tonic STING activity is low; however, uptake of tumor-derived cGAMP or direct sensing of micronuclear DNA rapidly activates the STING–TBK1–IRF3 cascade, resulting in autocrine/paracrine IFN-β production.^37^ This IFN-β acts in three complementary ways: (1) it drives STAT1-dependent reprogramming from an M2-like to an M1-like phenotype characterized by high IL-12, CXCL9/10 and nitric oxide, thereby restoring tumoricidal activity^9^; (2) it up-regulates MHC-II and CD80/86, converting TAMs into effective antigen-presenting cells that prime de-novo CD8⁺ T-cell responses;^38^ and (3) it creates a chemokine gradient that recruits granzyme-B⁺ CD8⁺ T and NK cells deep into the tumor bed. Importantly, myeloid-specific deletion of the negative regulator USP18 or PP2Ac/STRN4 further amplifies this IFN-I loop, leading to marked expansion of central-memory CD8⁺ T cells and synergistic efficacy with anti-PD-1.^39^ Conversely, antibody-mediated blockade of IFNAR1 on TAMs abrogates the therapeutic benefit of STING agonists, confirming that macrophage-intrinsic type-I IFN signaling is indispensable for sustained anti-tumor immunity. Collectively, these data establish TAM-derived type-I IFN as a master switch that re-wires myeloid–T-cell crosstalk and underpins the rationale for combination strategies that unleash this pathway in ccRCC.

Although ENPP1 has been reported to hydrolyze extracellular cGAMP in breast cancer and glioblastoma, our integrated analyses exclude any meaningful contribution of ENPP1 in ccRCC. ENPP1 and ENPP3 exhibit distinct physiologic expression patterns: ENPP1 is most abundant in the liver, bone, adipose and vascular endothelium, whereas ENPP3 is restricted to the intestine, kidney, placenta and specific immune subsets. This tissue segregation is preserved in malignancy. TCGA profiling confirms negligible ENPP1 transcripts in ccRCC, contrasting with high ENPP3 levels that correlate tightly with HIF-1α activation. Immunostaining of consecutive patient specimens confirmed undetectable ENPP1 protein, while ENPP3 was abundant on both tumor cells and peri-tumoral stroma.^10^ Consequently, the hypoxic renal microenvironment selectively licenses ENPP3, not ENPP1, to dominate extracellular cGAMP hydrolysis and thereby disable STING-dependent anti-tumor immunity.

In summary, we establish ENPP3 as a hypoxia-driven, HIF-1α–dependent checkpoint that is selectively over-expressed in ccRCC and functionally hijacks the cGAS–STING axis by hydrolyzing extracellular cGAMP. Genetic or antibody-mediated blockade of ENPP3 reinstates a TAM-restricted type-I interferon response, unleashes the CXCL9/10- and CCL5-mediated recruitment of cytotoxic T and NK cells, and triggers durable tumor regression without systemic toxicity. These data provide the first mechanistic link between chronic tumor hypoxia, aberrant nucleotide signaling, and primary immune evasion in ccRCC, and nominate ENPP3 as a readily druggable target whose inhibition can convert an immune-excluded “cold” tumor into an inflamed, anti-PD-1-responsive micro-environment. Clinically, ENPP3 abundance may serve as a predictive biomarker for response to combination regimens combining ENPP3-neutralizing antibodies, STING agonists, or immune-checkpoint inhibitors, offering a precision therapeutic avenue for the 60%–70% of ccRCC patients who currently fail front-line immunotherapy.

## Ethics statement

Approval of the research protocol by an Institutional Review Board: Hospital Review Board of the Second Affiliated Hospital of Anhui Medical University (3204-2024-F3).

Informed Consent. N/A.

Registry and the Registration No. of the study/trial. N/A.

Animal Studies. Approved by the IACUC of the Second Affiliated Hospital of Anhui Medical University.

## Consent for publication

All the authors are aware of and agree to the content of the paper and their authorships.

## Data Availability

All data generated or analysed during this study are included in this published article.
